# Validation of a Proteomic Signature of Lung Cancer Risk from Bronchial Specimens of Risk-Stratified Individuals

**DOI:** 10.3390/cancers15184504

**Published:** 2023-09-10

**Authors:** S.M. Jamshedur Rahman, Sheau-Chiann Chen, Yi-Ting Wang, Yuqian Gao, Athena A. Schepmoes, Thomas L. Fillmore, Tujin Shi, Heidi Chen, Karin D. Rodland, Pierre P. Massion, Eric L. Grogan, Tao Liu

**Affiliations:** 1 Division of Allergy, Pulmonary and Critical Care Medicine, Vanderbilt-Ingram Cancer Center, Vanderbilt University Medical Center, Nashville, TN 37232, USA; j.rahman@vumc.org (S.M.J.R.); pierre.massion@vumc.org (P.P.M.); 2 Department of Biostatistics, Vanderbilt University, Nashville, TN 37203, USA; sheau-chiann.chen.1@vumc.org (S.-C.C.); heidi.chen@vumc.org (H.C.); 3 Biological Sciences Division, Pacific Northwest National Laboratory, Richland, WA 99354, USA; wetproteome@gmail.com (Y.-T.W.); yuqian.gao@pnnl.gov (Y.G.); athena.schepmoes@pnnl.gov (A.A.S.); tujin.shi@pnnl.gov (T.S.); 4 Environmental Molecular Sciences Division, Pacific Northwest National Laboratory, Richland, WA 99354, USA; thomas.fillmore@pnnl.gov; 5 Department of Cell, Developmental, and Cancer Biology, Oregon Health and Science University, Portland, OR 97201, USA; rodland@ohsu.edu; 6 Veterans Affairs Tennessee Valley Healthcare System, Nashville, TN 37232, USA; 7 Department of Thoracic Surgery, Vanderbilt University Medical Center, Nashville, TN 37232, USA

**Keywords:** early detection, lung cancer, high risk, proteomics, bronchial epithelium

## Abstract

**Simple Summary:**

Lung cancer remains one of the deadliest cancers, with the worst survival rate. Cells from the airway lining of patients at high risk for lung cancer differentially express proteins that may serve as biomarkers for early detection of lung cancer. Previously, we identified candidate proteins in patients at high risk for lung cancer. In the present study, we validated several proteins in an independent cohort of 179 patients. Patients without lung cancer were stratified at low and high risk using an established risk model. Several proteins were found to be significantly overexpressed in the normal airways of individuals at high risk for lung cancer compared to the low-risk group. Our goal is to deliver a signature of risk that may provide the basis for lung cancer risk assessment and possibly novel future prevention strategies.

**Abstract:**

A major challenge in lung cancer prevention and cure hinges on identifying the at-risk population that ultimately develops lung cancer. Previously, we reported proteomic alterations in the cytologically normal bronchial epithelial cells collected from the bronchial brushings of individuals at risk for lung cancer. The purpose of this study is to validate, in an independent cohort, a selected list of 55 candidate proteins associated with risk for lung cancer with sensitive targeted proteomics using selected reaction monitoring (SRM). Bronchial brushings collected from individuals at low and high risk for developing lung cancer as well as patients with lung cancer, from both a subset of the original cohort (batch 1: n = 10 per group) and an independent cohort of 149 individuals (batch 2: low risk (n = 32), high risk (n = 34), and lung cancer (n = 83)), were analyzed using multiplexed SRM assays. ALDH3A1 and AKR1B10 were found to be consistently overexpressed in the high-risk group in both batch 1 and batch 2 brushing specimens as well as in the biopsies of batch 1. Validation of highly discriminatory proteins and metabolic enzymes by SRM in a larger independent cohort supported their use to identify patients at high risk for developing lung cancer.

## 1. Introduction

A major challenge in lung cancer prevention and cure hinges on identifying at-risk populations that ultimately develop lung cancer. Discovering molecular alterations in the bronchial epithelium of individuals at high risk for lung cancer is one of the minimally invasive procedures that has the potential to diagnose lung cancer at the earliest phase of lung tumorigenesis. Among the existing candidate proteomic biomarkers, only a few are authorized for use, but none of these have passed the clinical utility trial. Genomic and mutational signatures in lung tumor tissues and bronchial epithelium have been reported [1,2,3]. Alongside the genomic risk stratification models, the use of proteomic biomarkers to identify individuals at high risk for developing lung cancer is warranted for increased surveillance strategies, as proteins are the functional elements that execute most of the biochemical reactions (e.g., differentially expressed proteins in the normal bronchial epithelium induced by e-cigarette vapors have been identified [4]). Our goal is to identify robust proteomic candidate biomarkers from the airway epithelium to identify high-risk individuals. Proteomic alterations take place in the normal-appearing bronchial epithelium, ultimately causing histological changes resulting in preneoplastic lesions [5,6]. Previously, we reported proteomic alterations in the cytologically normal bronchial epithelial cells collected from the bronchial brushings of individuals at risk for lung cancer. We identified proteins by shotgun proteomics using liquid chromatography-tandem mass spectrometry (LC-MS/MS) and validated selected candidate biomarkers of risk in an independent cohort by parallel reaction monitoring [7]. These alterations associated with increasing risk were dominated by the overexpression of metabolic enzymes, particularly enzymes implicated in the Warburg effect and tumorigenesis. To our knowledge, there is no report describing proteomic dysregulation in bronchial epithelial cells from high-risk patients prior to the development of overt lung cancer.

The purpose of the current study is to validate, in independent cohorts from the Vanderbilt University Medical Center and Nashville Veteran Affairs Medical Center, a selected list of 55 candidate proteins (Table 1) associated with risk for lung cancer with a sensitive and precise targeted proteomics method using selected reaction monitoring (SRM). Orthogonal validation of promising biomarker candidates in independent patient cohorts, using a technically distinct measurement platform, is widely considered an essential step prior to full-scale clinical trials of novel biomarkers. MS-based proteomics has been extensively used to identify and profile proteins in biofluids and tissues. Antibody-free, MS-based measurement of proteins has demonstrated promise in the targeted quantification of candidate biomarkers [7]. Multiplexed, targeted proteomics using SRM provides the necessary sensitivity, specificity, and throughput for simultaneous validation of large sets of candidate biomarkers [8]. In this study, about half of the selected proteins are metabolic enzymes that demonstrated significant trends in our previous study. Proteins were analyzed in the bronchial specimens collected from two batches of patients, including batch 1 (n = 30; Table 2), which is a subset of the previously studied cohort [7], and a completely independent new cohort, batch 2 (n = 149; Table 3). The risk levels of the patients without lung cancer were determined using the Tammemagi risk model [9] as low risk and high risk for developing lung cancer.

## 2. Materials and Methods

### 2.1. Patient Cohorts and Collection of Bronchial Specimens

Approval for the conduct of this study was obtained from the Institutional Review Boards of the Vanderbilt University Medical Center, the Nashville Veteran Affairs Medical Center, and the Pacific Northwest National Laboratory in accordance with federal regulations. Bronchial specimens were collected at the time of bronchoscopy from the main stem bronchus of two batches of consenting never-smoker, former smoker, and current smoker patients at the Vanderbilt University Medical Center and Nashville Veteran Affairs Medical Center. Patients without lung cancer were stratified into low-risk and high-risk groups based on the Tammemagi risk model [9]. Patients with 2% or less risk were grouped as low-risk, and those with 3% or more risk were grouped as high-risk. Bronchial specimens were also collected from patients with lung cancer. In the first batch (batch 1, n = 30), there were 10 patients in each of the low-risk, high-risk, and lung cancer groups. Bronchial brushings and matched bronchial biopsies were collected from each patient in batch 1. The second batch (batch 2, n = 149) consisted of 32 low-risk, 34 high-risk, and 83 lung cancer patients. Only bronchial brushings but not bronchial biopsies were used in batch 2. Characteristics of the patients in batches 1 and 2 are shown in Table 2 and Table 3, respectively. Characteristics of individual volunteers are shown in Table S1. The specimen collection procedures were described in our previous publications [7,10]. Briefly, bronchial biopsies were snap frozen, and bronchial brushings were collected in normal saline on ice. Bronchial epithelial cells were dislodged from the brushes by low-speed vortexing and centrifugation at 300× *g* for 5 min. Bronchial biopsies and bronchial epithelial cells, after removing the supernatant, were stored at −80 °C freezer. Bronchial brushings consisted of over 95% normal bronchial epithelial cells, as reported in our previous study [7].

### 2.2. Tryptic Digestion of Proteins

For brushing specimens with an initial volume greater than 500 µL, the samples were initially concentrated using an Amicon 0.5-mL 3K molecular weight cutoff concentrator (EMD Millipore, Billerica, MA), and a buffer exchange into 50 mM NH_4_HCO_3_ (Sigma Aldrich, St. Louis, MO, USA) was also performed. The samples were sonicated for 30 s and chilled on ice for 30 s. This sonication/chill cycle was repeated for a total of 3 min. Then, a BCA Protein Assay (Thermo Fisher Scientific, Waltham, MA, USA) was performed, and a 100 µg aliquot was removed for digestion.

2,2,2-Trifluoroethanol (TFE, Sigma Aldrich) was added to reach a final concentration of 50% by volume. The samples were incubated at 60 °C for 2 h. Then, dithiothreitol (DTT, Sigma Aldrich) was added to have a final concentration of 2 mM. The samples were incubated for 1 h at 37 °C. Next, the samples were diluted 5× with Milli-Q H_2_O (EMD Millipore) before adding sequencing-grade modified trypsin (Promega, Madison, WI, USA). Trypsin was added in a 1:50 enzyme/protein ratio. The samples were incubated for 3 h at 37 °C.

Following the trypsin digestion, the samples were frozen in liquid nitrogen and stored at −80 °C. A solid-phase extraction (SPE) was performed using 50 mg, 1 mL C18 SPE cartridges (Phenomenex, Torrance, CA, USA). The eluted peptides were concentrated in the Speed-Vac and a final BCA protein assay was performed. For the batch 2 samples, the LC-SRM sample loading was based on the BCA measurement after SPE cleanup. In batch 1, an additional BCA protein assay was performed before the SPE cleanup, and the LC-SRM sample loading was based on the BCA measurement before the SPE cleanup.

### 2.3. SRM Assay Development

For SRM measurement of the targeted peptides, stable isotope-labeled peptides (SI peptides) (crude grade) were synthesized (New England Peptide, Gardner, MA, USA) with ^13^C/^15^N on the C-terminal lysine (K) and arginine (R). The SI peptides were dissolved in 15% acetonitrile (ACN) and 0.1% formic acid (FA) at a concentration of 2 mM and stored at −80 °C. A mixture of these SI peptides was made with a final concentration of 5 pmol/μL for each peptide.

The mixture of SI peptides was analyzed by LC–MS/MS using Orbitrap Fusion Lumos (HCD mode), and the Thermo RAW files were processed with DTARefinery [11] (v1.2) and MS-GF+ [12,13] (v9881) to match against the RefSeq human protein sequence database, released on 18 April 2017 (hg19; 46,174 proteins), combined with 245 contaminants (e.g., trypsin, keratin) for generating a list of MS/MS fragment ions derived from SI peptides. Then, the peptide list was analyzed using Skyline software (Version 21.2) to build the peptide library and output the ion transition list for LC-SRM. Three or more MS/MS fragment ions were selected for SRM transitions of each targeted peptide based on strong ion intensity and at least three amino acids in the transition ions (see Table S2 for the final transition list).

### 2.4. LC-SRM Analysis

The SI peptides were spiked in the peptide samples, and the batch 1 and batch 2 samples were analyzed using a TSQ Vantage and a TSQ Altis triple quadruple mass spectrometer (Thermo Fisher Scientific), respectively. Both instruments were equipped with a nanoACQUITY UPLC system (Waters, Milford, MA, USA).

For batch 1, the MS parameters were set as follows: 0.7 fwhm Q1 and Q3 resolution, 1.0 s cycle time, and 1.5 mTorr gas pressure. Collision energy (CE) was derived from Skyline software (Version 21.2) for every SRM transition. Data were acquired in a time-scheduled SRM mode with a retention time window of 4 min. The LC used a 75 µm i.d. × 20 cm, BEH 1.7 µm C18 capillary column (Waters) and performed a 100 min gradient with a 300 nL/min flow rate at a temperature of 44 °C. The mobile phases were (A) 0.1% FA in water and (B) 0.1% FA in ACN, and the gradient profile was as follows (min: %B): 0:1, 10:5, 70:30, 80:90, 83:90, 85:1, and 100:1.

For batch 2, the MS parameters were set as follows: 0.7 fwhm Q1 and Q3 resolution, 1.05 s cycle time, and 1.5 mTorr gas pressure. Data were acquired in a time-scheduled SRM mode with a retention time window of 35 min. The LC used a 100 µm i.d. × 10 cm, BEH 1.7 µm C18 capillary column (Waters) and performed a 110 min gradient with a 400 nL/min flow rate at a temperature of 44 °C. The mobile phases were the same as those in batch 1, and the gradient profile was as follows (min: %B): 7:1, 9:6, 40:13, 70:22, 80:40, 85:95, 93:50, 94:95, 95:1, and 110:1.

### 2.5. SRM Data Analysis

SRM data were analyzed using the Skyline software [14] (Version 21.2). The total peak area ratios of endogenous light peptides and their heavy isotope-labeled internal standards (i.e., L/H peak area ratios) were exported for quantitation (Table S2). Peak detection and integration were carried out according to two criteria: (1) the same retention time and (2) similar peak area ratios for the transitions. All data were manually inspected to ensure correct retention time, peak detection, and accurate integration. SRM data were generated for two surrogate peptides for most of the protein candidates where feasible, and the L/H peak area ratios of the most detectable peptide were used for statistical analysis (Table S2). For batch 2, the L/H peak area ratios exported from Skyline were used directly for subsequent statistical analysis. For batch 1, the ratios between the BCA assay results before and after SPE cleanup were used to normalize the L/H peak area ratios exported from Skyline, and the normalized data were used for subsequent analysis. All SRM data are organized as Skyline files on the Panorama server [15] via https://panoramaweb.org/Signature_of_lung_cancer_risk.url (accessed on 6 September 2023).

### 2.6. Statistical Analysis

Descriptive statistics were summarized for demographic, clinical, and protein data using the mean/median (St.Dev./Range) for continuous variables and frequencies (percentages) for categorical variables. Between-group differences were assessed using the Wilcoxon rank-sum test for continuous variables and Pearson’s chi-square or Fisher’s exact test for categorical variables. The relationship between protein and risk score was also evaluated using Spearman’s rank correlation coefficient (r) with a 95% confidence interval (CI). To deal with multicollinearity among proteins, penalized regression with least absolute shrinkage and selection operator (LASSO) regularization [16] was performed. The penalized logistic regression was used for a binary outcome variable (i.e., high risk vs. low risk), and the penalized linear regression was used for a continuous outcome variable (i.e., risk score). Both penalized regression models used the LASSO regulation and would select the proteins that were the most important variables with the least correlated between proteins. The area under the curve (AUC) was used to evaluate the discrimination of the penalized logistic regression model. The mean square error (MSE) was used to measure the difference between actual and estimated risk score values in the penalized linear regression model. Internal validation with bootstrap 0.632 was also performed for reporting the optimism-adjusted AUC. A Venn diagram was used to illustrate the relationship between two or more concepts/groups. A two-sided p-value less than 0.05 is statistically significant.

## 3. Results

### 3.1. Candidate Protein Selection for Validation

Previously, we reported proteomic signatures for the early detection of lung cancer [7]. In the current study, we selected 55 candidate proteins, 44 of which were selected for validation from our previously published study with bronchial brushing specimens [7]. Eleven more candidate proteins were selected based on their promise as potential biomarkers for the early detection of lung cancer. Among the 55 selected candidates, 25 are metabolic enzymes, 17 are signaling proteins with diverse functions, three are DNA repair proteins, two are oncoproteins, two are histone methylation enzymes, and there is one each of galactose binding protein, oncoprotein and transcription factor, transcription factor, serine proteinase inhibitor, pulmonary surfactant protein, and ubiquitin conjugation enzyme (Table S3). Carbohydrate and lipid metabolic dysregulation in cancer cells is well known, and we reported similar dysregulation in histologically normal bronchial epithelial cells [7]. In the current study, twenty-one selected glucose and fatty acid metabolic enzymes are ACLY, ACSF3, ALOX15, DLST, FASN, G6PD, GLB1, GLUD1, IDH1, IDH2, LDHA, LDHB, ME2, PFKL, PFKP, PGD, PGK1, PGM1, PKM2, PYGB, and UGP2. Metabolic enzymes AKR1B10, ALDH3A1, and ALDH1A1 have been reported by our group and others as leading candidate biomarkers for multiple cancer types, including lung [7]. The trifunctional folate metabolic enzyme MTHFD1 also resides in the nucleus and is recruited for transcriptional regulation [17]. Histone methyltransferase KMT2D (also called MLL4) was added to the list and tested in batch 2 only because it has recently been found to be a tumor suppressor and frequently mutated in lung cancer [18]. It is among the most highly inactivated epigenetic modifiers in lung cancer [19]. The carcinoembryonic antigen family member, CEACAM5, is being used as a clinical cancer biomarker and plays a role in cell adhesion, signaling, and tumor progression [20,21].

### 3.2. Validation of the Dysregulated Proteins

**Figure 1:**
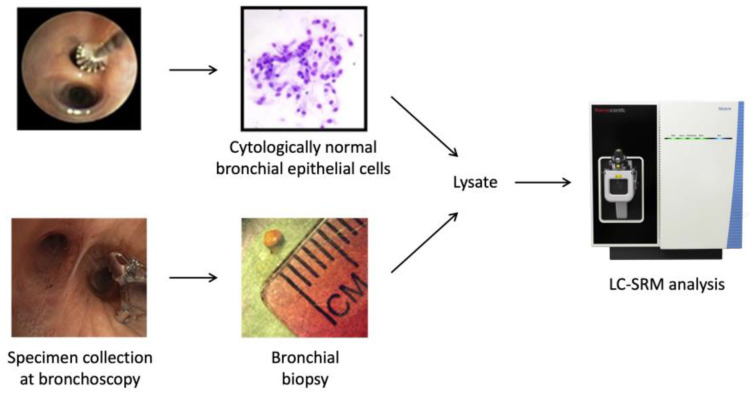
Schematic workflow. Bronchial specimens were collected at the time of bronchoscopy from the main stem bronchus, followed by proteomics sample processing (digestion and cleanup) and multiplexed targeted proteomics analysis of the candidate proteins using LC-SRM.

**Figure 2:**
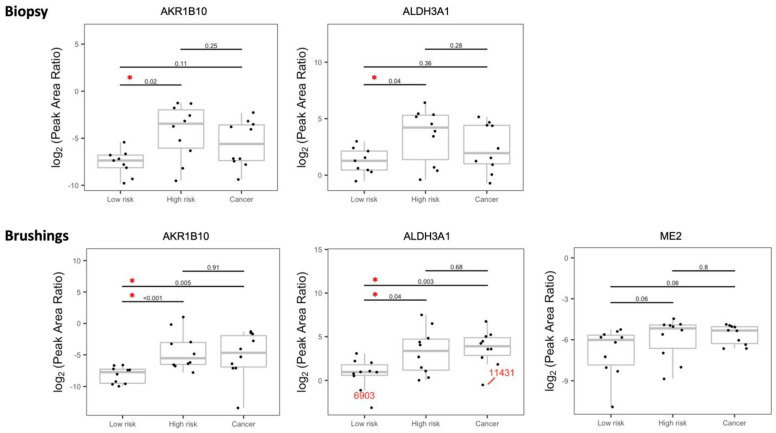
Boxplot with dots of SRM peak area ratio (endogenous/IS) on a log_2_ scale by risk/cancer group in the bronchial biopsies (the top panels) and bronchial brushings (the bottom panels) of patients from batch 1. Differential expressions of ALDH3A1 and AKR1B10 between low-risk and high-risk groups are statistically significant in both the bronchial biopsies and bronchial brushings from batch 1 patients. The biopsies and brushings were collected from the same patient. Comparisons between samples from patients with lung cancer and risk groups are also presented. All candidate proteins were measured in the same run using LC-SRM with heavy isotope-labeled synthetic surrogate peptides as internal standards. Red asterisk indicates *p*-value for testing the difference comparison between groups < 0.05. Sample ID in red color: any observations that are more than 1.5 interquartile range (IQR) below Q1 or more than 1.5 IQR above Q3.

Both the bronchial brushings and bronchial biopsies were analyzed by LC-SRM. Figure 1 shows the schematic workflow. Bronchial specimens were collected at the time of bronchoscopy from the main stem bronchus. Bronchoscopy and bronchial epithelial cells were lysed to extract proteins for trypsin digestion. Stable isotope-labeled peptides of target proteins were spiked into individual samples, and LC-SRM analysis was performed. Endogenous and SI peptide peak areas were obtained for relative quantification across the samples. In batch 1, where 54 proteins were tested, 49 proteins were detected in the bronchial biopsies and 44 proteins were detected in the bronchial brushings. Overall, higher variability was observed among the biopsies compared to the brushings, which may be attributed to the tissue heterogeneity in the bronchial biopsies. The percentage of bronchial epithelium in the biopsy samples was highly variable, whereas over 90% of the cells in the bronchial brushings are epithelial cells [7]. We observed significant overexpression of AKR1B10 and ALDH3A1 in both biopsies and brushings of the high-risk group compared to the low-risk group (Figure 2). In the brushings, ME2, a tricarboxylic acid cycle (TCA) enzyme, marginally missed statistical significance (*p* = 0.06).

**Figure 3:**
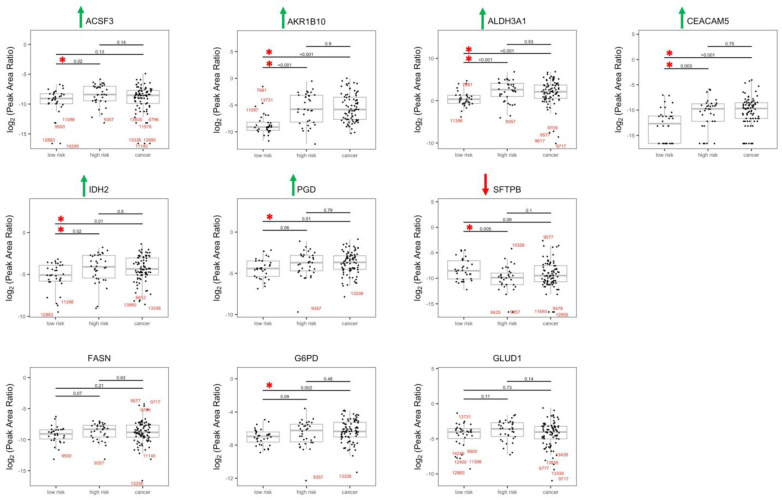
Boxplot with dots of SRM peak area ratio (endogenous/IS) on a log_2_ scale by risk/cancer group in the bronchial brushings of patients from batch 2. Differential expressions of candidate proteins between low-risk and high-risk groups in the bronchial brushings of patients from batch 2 are shown. Comparisons between samples from patients with lung cancer and risk groups are also presented. All candidate proteins were measured in the same run using LC-SRM with heavy isotope-labeled synthetic surrogate peptides as internal standards. Red asterisk indicates *p*-value for testing the difference comparison between groups < 0.05. Sample ID in red color: any observations that are more than 1.5 IQR below Q1 or more than 1.5 IQR above Q3. Green arrows: up-regulation of protein. Red arrow: down-regulation of protein.

To validate the selected proteins in batch 1, univariate analysis was used to explore each variable in batch 2; KMT2D was tested only in batch 2. Out of 55 proteins, 51 were detected in the bronchial brushing samples, and 8 out of 51 proteins were significantly dysregulated when compared between low-risk and high-risk groups in batch 2 (Figure 3). Significant upregulation of ACSF3, AKR1B10, ALDH3A1, CEACAM5, and IDH2 in the high-risk group was validated, and SFTPB was the only protein that was downregulated in the high-risk group compared to the low-risk group. Downregulation of SFTPB was also observed in our previous study [7] as well as in a recent study by Wang et al. [22]. Although upregulation of FASN, GLUD1, and G6PD in the high-risk group barely missed statistical significance, these enzymes deserve attention given their biological significance in cancer and metabolism (Figure 3, Table S3). Importantly, AKR1B10 and ALDH3A1 were significantly overexpressed in both batches as well as in biopsies in batch 1. To our knowledge, this is the first study where both brushings and biopsies from low-risk and high-risk individuals who do not have lung cancer were analyzed. The fact that the results from both types of specimens mostly concurred is reassuring.

Expression levels of the proteins were not significantly different between the high-risk group and the cancer group except for LGALS7B in batch 1 and KMT2D in batch 2. Also, when multivariable analysis was performed to distinguish patients with high-risk from cancer patients, only LEG7 was selected in batch 1, and ACSF3, FXR1, KMT2D, LDHA, ME2, MTHFD1, and SFTPB were chosen in batch 2. While this comparison is important, our main focus was on distinguishing between the high-risk and low-risk groups.

**Figure 4:**
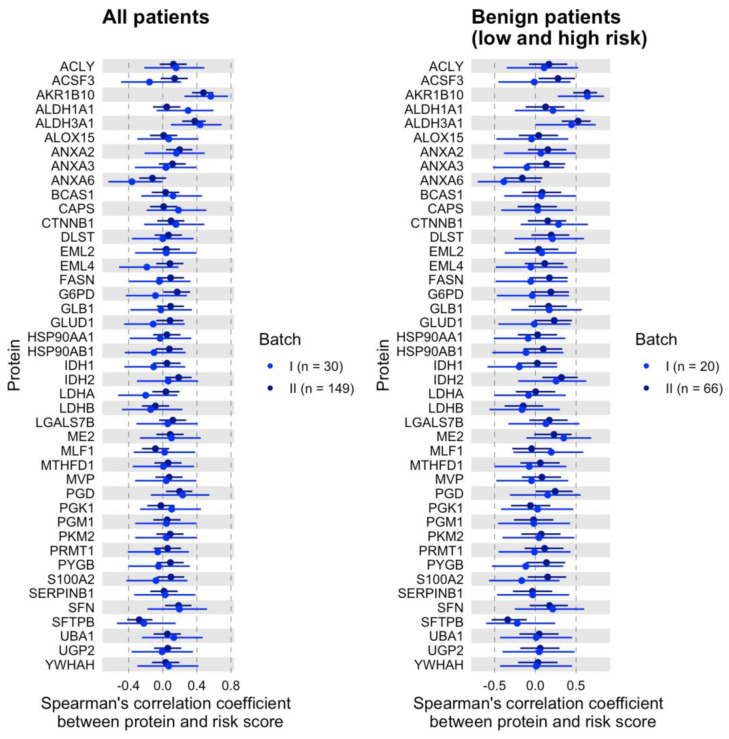
Spearman’s rank correlation coefficient (blue dot) with 95% confidence interval (line) between each protein and risk score stratified by batch. (**Left**) all patients; (**Right**) benign patients, including low- and high-risk scores.

Furthermore, for each batch, the correlation between each protein and risk score was evaluated in all patients and subgroups (low- and high-risk groups), as shown in Figure 4. AKR1B10 and ALDH3A1 are significantly correlated with a risk score for batch 1 (AKR1B10: *r* with 95% CI = 0.563 (0.254, 0.767), *p* = 0.001; ALDH3A1: *r* with 95% CI = 0.440 (0.095, 0.691), *p* = 0.015) and batch 2 (AKR1B10: *r* with 95% CI = 0.478 (0.344, 0.593), *p* < 0.001; ALDH3A1: *r* with 95% CI = 0.375 (0.228, 0.505), *p* < 0.001). The two proteins were also found in subgroup correlation analysis in batch 1 (AKR1B10: *r* with 95% CI = 0.639 (0.275, 0.843), *p* = 0.002; ALDH3A1: *r* with 95% CI = 0.444 (0.002, 0.741), *p* = 0.05) and batch 2 (AKR1B10: *r* with 95% CI = 0.635 (0.464, 0.76), *p* < 0.001; ALDH3A1: *r* with 95% CI = 0.525 (0.324, 0.681), *p* < 0.001).

### 3.3. Dysregulated Proteins Discriminate High-Risk Groups from Low-Risk Groups

**Figure 5:**
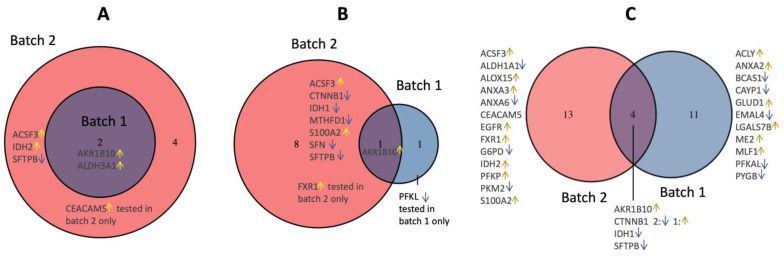
Venn diagrams for the similarities and differences between the selected proteins from batch 1 and batch 2 (subgroup: high- and low-risk groups) across three different statistical methods. (**A**) Wilcoxon rank-sum test (high vs. low); (**B**) Logistic regression with lasso regularization for a binary outcome (high vs. low); (**C**) Linear regression with lasso regularization for a continuous outcome (risk score) where the data were a subset of patients with benign nodules only. The yellow arrow indicates up-regulation (the definition here is that the high-risk group has a higher level of the protein than the low-risk group); the blue arrow indicates down-regulation.

To distinguish individuals at low risk from those at high risk, AKR1B10 and ALDH3A1 demonstrated strong discriminatory power in both batch 1 and batch 2. Four more proteins, ACSF3, CEACAM5, IDH2, and SFTPB, were discriminatory between the two groups in batch 2 (Figure 5A). Next, we compared the proteins selected from multivariable regression with LASSO regularization (Figure 5B) to the results using univariate analysis (Figure 5A). In batch 1, PFKL was added instead of ALDH3A1; in batch 2, CTNNB1, IDH1, MTHFD1, S100A2, and SFN were selected, but IDH2, CEACAM5, and ALDH3A1 in Figure 5A were excluded in Figure 5B. Thus, the upregulation of AKR1B10 in the cytologically normal bronchial epithelium is an overlap predictor of the risk of developing lung cancer (Figure 5B). When the risk score is fitted as a continuous outcome instead of a binary outcome in the multivariable model, CTNNB1, IDH1, and SFTPB came out as additional predictors in both batches (Figure 5C).

**Figure 6:**
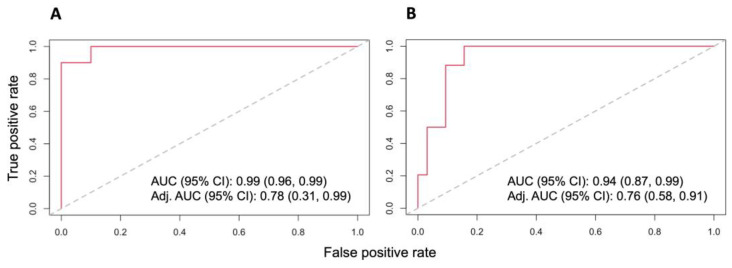
Receiver operating characteristic (ROC) curve for evaluating the performance of separating the low-risk from high-risk groups based on the lasso logistic regression with (**A**) the important proteins AKR1B10 and PFKL in batch 1; and (**B**) the important proteins ACSF3, AKR1B10, CTNNB1, FXR1, IDH1, MTHFD1, S100A2, SFN, and SFTPB in batch 2. AUC is the area under the curve; CI is the confidence interval; Adj. AUC is the adjusted AUC for optimism.

In addition, we measured the discriminatory power of the binary logistic regression model with lasso regulation. For high-risk group vs. low-risk group, the original area under curve (AUC) with 95% CI is 0.99 (0.96, 0.99) and the optimism-adjusted AUC is 0.78 (0.31, 0.99) in batch 1 (Figure 6A). The original AUC with 95% CI is 0.94 (0.87, 0.99) and the optimism-adjusted AUC with 95% CI is 0.76 (0.58, 0.91) in batch 2 (Figure 6B).

**Figure 7:**
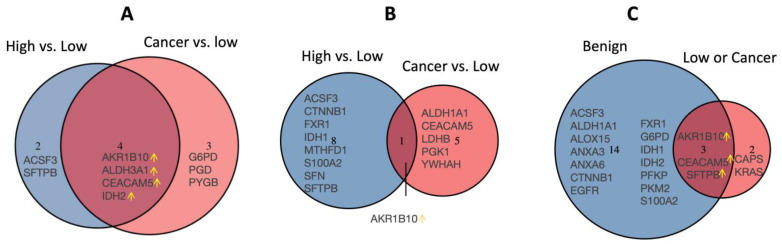
Venn diagrams for the similarities and differences between the selected proteins from high vs. low groups and those from cancer vs. low groups within batch 2 across three different statistical methods. (**A**) Wilcoxon rank-sum test; (**B**) Logistic regression with lasso regularization for a binary outcome (high vs. low or cancer vs. low); (**C**) Linear regression with lasso regularization for a continuous outcome (risk score) where “benign” was a subset of patients with benign nodules; “low or cancer” were a subset of patients with low-risk or cancer nodules. The yellow arrow indicates up-regulation where the low-risk group is set as the reference level of the group variable for the two comparisons (high vs. low and cancer vs. low), i.e., the up-regulation proteins (shown in the overlap of the Venn diagrams) were higher in the cancer or high-risk group than in the low-risk group.

Since none of the proteins were differentially expressed between the high-risk and lung cancer groups for univariate analysis (Figure 2 and Figure 3), we sought to refine, by use of a Venn diagram (Figure 7A), the proteins that discriminate the low-risk group from the high-risk and lung cancer groups from the results of the larger batch 2 cohort. In the univariate analysis, compared to the low-risk group, AKR1B10, ALDH3A1, CEACAM5, and IDH2 are higher than the high-risk or cancer group, i.e., their upregulation discriminates the low-risk group from the high-risk group as well as the lung cancer group. When the most significant variables are kept in the LASSO regression, AKR1B10 remain the discriminatory proteins (Figure 7B). When the original risk score was analyzed as a continuous outcome in the benign (low and high) subgroup as well as the “low and cancer” subgroup, the common proteins between the two subgroups, SFTPB and CEACAM5, were added to AKR1B10 to explain the variation of the risk scores (Figure 7C). Overall, these results demonstrate the robustness of AKR1B10 and CEACAM5 in the cytologically normal bronchial epithelium in discriminating both the high-risk and lung cancer groups from the low-risk group.

## 4. Discussion

We present the results of the validation of candidate proteomic biomarkers that discriminate patients at low and high risk for developing lung cancer with high accuracy. The published reviews from our group emphasized the importance of narrowing down high-risk individuals with incidental lung nodules [23]. In the current study, we used bronchial epithelial cells from bronchial brushing specimens collected by a semi-invasive bronchoscopy procedure from patients who did not have lung cancer. We used the Tammemagi risk model [9] to stratify patients without lung cancer as low-risk and high-risk for developing lung cancer. The focus of this study was to validate and refine proteomic biomarkers from a selected list of candidate proteins that discriminate the high-risk group from the low-risk group.

The potential clinical utility of these proteomic markers lies in the improved stratification of patients with indeterminate lung nodules identified on screening studies. Identifying low-risk individuals would save them from going through unnecessary procedures, expenses, and anxieties, while identification of patients at high risk could trigger increased surveillance, thus potentially improving clinical outcome. The fact that the expression of ALDH3A1 and AKR1B10 in the high-risk group is not significantly different from the lung cancer group but significantly different from the low-risk group in both biopsies and brushings in batch 1 and in the brushings in batch 2 means that it can be used to both rule out low-risk individuals from surveillance but also flag some patients from the high-risk groups with confidence for greater surveillance procedures (Figure 2 and Figure 3). In batch 2, CEACAM5 and IDH2 demonstrated identical results in the brushings, and as expected, the low-risk group is significantly different from the lung cancer group. Consistent with the previously published studies, significant downregulation of SFTPB in the high-risk group compared to the low-risk group highlights its discriminatory potential in the cytologically normal bronchial epithelium (Figure 3).

Despite the discovery of a large number of proteomic biomarkers, very few of these biomarkers have been validated or used in clinical practice. The challenge is the statistical analysis of relatively large sets of peptides/proteins from LC-MS proteomic data from relatively smaller sample cohorts [24]. The identification of many metabolic enzymes whose expression levels changed led us to focus on metabolic reprogramming in our previous study [7]. Corroborating our earlier reports, some of the key metabolic enzymes, AKR1B10, ALDH3A1, ACSF3, IDH2, ME2, CEACAM1, and SFTPB, were validated in the current study. It is likely that in batch 1, the limited sample number might have precluded the statistical significance of some of the key enzymes that were validated in batch 2. Therefore, the above metabolic enzymes and their relevant pathways deserve attention in future validation strategies.

Of particular interest, AKR1B10 (Aldosterone reductase family 1 member B10) was highly discriminatory between low-risk and high-risk groups in both batch 1 and batch 2. We observed upregulation of this aldo-keto reductase in the cytologically normal bronchial epithelium of patients at high risk for lung cancer by every statistical analytical method we used. AKR1B10 is a NAD(P)H-dependent oxidoreductase catalyzing interconversions between carbonyl and alcohol groups of endogenous xenobiotic compounds [25]. AKR1B10 limits the toxic side effects of oxidative stress as a result of increased utilization of fatty acid oxidation [26], which may protect cytologically normal bronchial epithelial cells during transformation. Such protective activity of ALDH3A1 is also essential for cell survival in response to both oxidative stress and lipid peroxidation [27]. It is interesting to note that both AKR1B10 and ALDH3A1 are upregulated in high-risk bronchial epithelial cells.

We observed variability in the results from biopsy specimens in batch 1, which could be attributed to the tissue heterogeneity in biopsies. Given the tiny size of the biopsies, Hematoxylin and Eosin staining of the biopsies was not performed. Therefore, we were unable to determine the contribution of the epithelium from each individual biopsy as well as demonstrate the degree of heterogeneity of the tissue composition of the biopsies. We used bronchial brushings only in the larger batch 2 cohort. Bronchial epithelial cells constitute over 95% of the bronchial brushing specimens [7].

## 5. Conclusions

In conclusion, validation of highly discriminatory proteins and metabolic enzymes by SRM in a larger independent cohort supported their use to identify patients at high risk for developing lung cancer. As described in the review from our group [23], these proteomic biomarkers in the bronchial brushing specimen may allow us to narrow down the high-risk individuals for increased surveillance. Future analysis using methods with increased sensitivity for the analysis of low-abundance protein candidates and carrier-assisted SRM approaches for the analysis of very small-sized samples would be the next step towards discovering and validating additional promising candidate biomarkers. Ultimately, this approach may deliver a signature of risk that may provide the basis for lung cancer risk assessment and possibly novel future prevention strategies.

## Figures and Tables

**Table 1:** Selected candidate proteins for validation.

Protein Symbol	Protein Name
ACLY	ATP citrate lyase
ACSF3	Acyl-CoA synthetase family member 3
AKR1B10	Aldo-keto reductase family 1, member B10 (aldose reductase)
ALDH1A1	Aldehyde dehydrogenase 1 family, member A1
ALDH3A1	Aldehyde dehydrogenase 3 family, memberA1
ALOX15	Arachidonate 15-lipoxygenase
ANXA2	Annexin A2
ANXA3	Annexin A3
ANXA6	Annexin A6
ATM	Ataxia telangiectasia mutated
ATR	Ataxia telangiectasia and Rad3 related
BCAS1	Breast carcinoma amplified sequence 1
BRCA2	Breast cancer type 2 susceptibility protein
CAPS	Calcyphosine
CEACAM5	Carcinoembryonic antigen-related cell adhesion molecule 5
CTNNB1	Catenin (cadherin-associated protein), beta 1, 88kDa
DLG5	Discs, large homolog 5 (Drosophila)
DLST	Dihydrolipoamide S-succinyltransferase
EGFR	Epidermal growth factor receptor
EML2	Echinoderm microtubule associated protein like 2
EML4	Echinoderm microtubule associated protein like 4
FASN	Fatty acid synthase
FXR1	Fragile X mental retardation, autosomal homolog 1
G6PD	Glucose-6-phosphate dehydrogenase
GLB1	Galactosidase, beta 1
GLUD1	Glutamate dehydrogenase 1
HSP90AA1	Heat shock protein 90 kDa alpha (cytosolic), class A member 1
HSP90AB1	Heat shock protein 90 kDa alpha (cytosolic), class B member 1
IDH1	Isocitrate dehydrogenase 1
IDH2	Isocitrate dehydrogenase 2
KMT2D	Histone-lysine N-methyltransferase 2D
KRAS	Kirsten rat sarcoma viral proto-oncogene
LDHA	Lactate dehydrogenase A
LDHB	Lactate dehydrogenase B
LGALS7B	Lectin, galactoside-binding, soluble, 7B
ME2	Malic enzyme 2, NAD(+)-dependent, mitochondrial
MLF1	Myeloid leukemia factor 1
MTHFD1	C-1-tetrahydrofolate synthase, cytoplasmic
MVP	Major vault protein
NFKB1	Nuclear factor of kappa light polypeptide gene enhancer in B-cells 1
PFKL	Phosphofructokinase, liver
PFKP	Phosphofructokinase, platelet
PGD	Phosphogluconate dehydrogenase
PGK1	Phosphoglycerate kinase 1
PGM1	Phosphoglucomutase 1
PKM2	Pyruvate kinase M2, muscle
PRMT1	Protein arginine methyltransferase 1
PYGB	Glycogen phosphorylase B
S100A2	S100 calcium binding protein A2
SERPINB1	Serpin peptidase inhibitor, clade B (ovalbumin), member 1
SFN	Stratifin
SFTPB	Surfactant protein B
UBA1	Ubiquitin-like modifier activating enzyme 1
UGP2	UDP-glucose pyrophosphorylase 2
YWHAH	14-3-3 protein eta

**Table 2:** Batch 1 patient characteristics.

Characteristics		Low Risk (n = 10)	High Risk (n = 10)	Lung Cancer (n = 10)
Age				
	Average + St.Dev.	61.8 ± 5.7	69.5 ± 4	73.5 ± 7.8
	Median (range)	60.5 (55–74)	69.5 (63–76)	76 (57–82)
Gender				
	Male	5 (50%)	9 (90%)	6 (60%)
	Female	5 (50%)	1 (10%)	4 (40%)
BMI				
	Average + St.Dev.	27.5 ± 6	27.8 ± 3.8	22.5 ± 1.9
	Median (range)	28 (19.7–37)	27.5 (21.3–32.7)	22.5 (19.3–25.8)
Smoking history				
	Never smoker	4 (40%)	0 (0%)	1 (10%)
	Former smoker	6 (60%)	4 (40%)	7 (70%)
	Current smoker	0 (0%)	6 (60%)	2 (20%)
	Pack year (Average + St.Dev.) *	30.9 ± 28.7	73.4 ± 30.1	51.2 ± 30.3
	Pack year (median)	23.5 (3.8–65)	59 (47–129)	45 (15–100)
COPD				
	Yes	0 (0%)	3 (30%)	4 (40%)
	No	10 (100%)	7 (70%)	6 (60%)
Histology				
	Adenocarcinoma	-	-	2 (20%)
	Squamous cell carcinoma	-	-	7 (70%)
	Small-cell lung cancer	-	-	1 (10%)
Path stage				
	No surgery			4 (40%)
	Tx Nx Mx	-	-	2 (20%)
	IA	-	-	1 (10%)
	IB	-	-	1 (10%)
	IIA	-	-	1 (10%)
	IV	-	-	1 (10%)

* Only former and current smokers.

**Table 3:** Batch 2 patient characteristics.

Characteristics		Low Risk (n = 32)	High Risk (n = 34)	Lung Cancer (n = 83)
Age				
	Average + St.Dev.	63.1 ± 10.1	65.7 ± 7.4	66.4 ± 6.3
	Median (range)	61 (43–87)	66 (48–87)	66 (53–85)
Gender				
	Male	14 (44%)	22 (65%)	56 (67%)
	Female	18 (56%)	12 (35%)	27 (33%)
BMI				
	Average + St.Dev.	27.9 ± 5.3	26.8 ± 5.2	27.1 ± 6.2
	Median (range)	27.6 (15.5–39.1)	26.1 (19.1–39)	26.6 (18.1–53)
Smoking history				
	Never smoker	10 (31%)	0 (0%)	1 (1%)
	Former smoker	19 (59%)	14 (41%)	36 (43%)
	Current smoker	3 (9%)	20 (59%)	46 (54%)
	Pack year (Average + St.Dev.)	14.9 ± 13.9	67.8 ± 25.7	53.2 ± 32.2
	Pack year (median)	18 (0–50)	61.5 (33–135)	50 (0–200)
COPD				
	Yes	7 (22%)	27 (79%)	61 (73%)
	No	25 (25%)	7 (21%)	22 (27%)
Histology				
	Adenocarcinoma	-	-	37 (45%)
	Squamous cell carcinoma	-	-	38 (46%)
	Adenosquamous cell ca.	-	-	1 (1%)
	Non-small-cell lung cancer	-	-	3 (3%)
	Large cell carcinoma	-	-	1 (1%)
	Large cell neuroendocrine	-	-	2 (2%)
	Small-cell lung cancer	-	-	1 (10%)
Path stage				
	Stage 0			1 (1%)
	IA	-	-	15 (18%)
	IB	-	-	11 (13%)
	IIA	-	-	1 (1%)
	IIB	-	-	9 (11%)
	IIIA	-	-	15 (18%)
	IIIB	-	-	6 (7%)
	IIIC	-	-	2 (2%)
	IVA	-	-	11 (13%)
	IVB	-	-	2 (2%)
	Limited	-	-	1 (1%)
	Unknown	-	-	9 (11%)

## Data Availability

All SRM data are organized as Skyline files on the Panorama server via https://panoramaweb.org/Signature_of_lung_cancer_risk.url (accessed on 6 September 2023).

## Supplementary Materials

The following supporting information can be downloaded at: https://www.mdpi.com/article/10.3390/cancers15184504/s1, Table S1: Individual patient characteristics; Table S2: SRM quantitation results; Table S3: Candidate protein information.
